# Effect of Glu-B3 Allelic Variation on Sodium Dodecyl Sulfate Sedimentation Volume in Common Wheat (*Triticum aestivum* L.)

**DOI:** 10.1155/2013/848549

**Published:** 2013-06-18

**Authors:** Hongqi Si, Manli Zhao, Fuxia He, Chuanxi Ma

**Affiliations:** ^1^School of Agronomy, Anhui Agricultural University, Hefei 230036, China; ^2^Key Laboratory of Wheat Biology and Genetic Breeding in Southern Huanghuai Wheat Region, Ministry of Agriculture, Hefei 230036, China; ^3^Anhui Key Laboratory of Crop Biology, Hefei 230036, China

## Abstract

Sodium dodecyl sulfate (SDS) sedimentation volume has long been used to characterize wheat flours and meals with the aim of predicting processing and end-product qualities. In order to survey the influence of low-molecular-weight glutenin subunits (LMW-GSs) at Glu-B3 locus on wheat SDS sedimentation volume, a total of 283 wheat (*Triticum aestivum* L.) varieties including landraces and improved and introduced cultivars were analyzed using 10 allele-specific PCR markers at the Glu-B3 locus. The highest allele frequency observed in the tested varieties was Glu-B3i with 21.9% in all varieties, 21.1% in landraces, 25.5% in improved cultivars, and 12% in introduced cultivars. Glu-B3 locus represented 8.6% of the variance in wheat SDS sedimentation volume, and Glu-B3b, Glu-B3g, and Glu-B3h significantly heightened the SDS sedimentation volume, but Glu-B3a, Glu-B3c, and Glu-B3j significantly lowered the SDS sedimentation volume. For the bread-making quality, the most desirable alleles Glu-B3b and Glu-B3g become more and more popular and the least desirable alleles Glu-B3a and Glu-B3c got less and less in modern improved cultivars, suggesting that wheat grain quality in China has been significantly improved through breeding effort.

## 1. Introduction

The end-use quality of bread wheat depends on the seed storage proteins. These proteins determine the strength and unique viscoelastic properties of the dough by adjusting the quantity and quality of the gluten formed. Low-molecular-weight glutenin subunits (LMW-GS) are redounded to dough extensibility and gluten strength [1]. Generally, the LMW-GS are encoded by gene families at the Glu-A3, Glu-B3, and Glu-D3 loci, located on the short arms of chromosomes 1A, 1B, and 1D, respectively. Based on their N-terminal amino acid sequences, LMW-GSs were classified as three subclasses: LMW-m, LMW-s, and LMW-i, which are named for the first amino acid residue of their mature proteins, methionine, serine, and isoleucine, respectively, [2]. The LMW-m type subunits can be divided into three subtypes, METSHIGPL-, METSRIPGL-, and METSCIPGL- [3]. Because cysteine residues form intramolecular and intermolecular disulfide bonds in the gluten macropolymer, previous researchers have classified the LMW-GSs into six types on the basis of the locations of cysteine residues [4]. Several LMW-GS genes have been isolated from bread wheat and its relatives [4, 5]. However, because of the lack of efficient methods to distinguish members of this complex, heterogeneic, and comigrating multigene family, the exact copy numbers of the LMW-GS genes are still unknown [2, 6]. SDS-PAGE, high-resolution capillary electrophoresis, reversed-phase high-performance liquid chromatography (RP-HPLC), matrix-assisted laser desorption/ionization time of flight (MALDI-TOF), two-dimensional gel electrophoresis (2DE), and mass spectrometry (MS) have been used to investigate the polymorphic LMW-GS complex in bread wheat [7–11]. However, the complex band/peak patterns of LMW-GSs and the overlapping mobility between LMW-GSs and gliadins posed a problem, particularly when testing allelic variations of LMW-GSs in various wheat varieties. High financial and labor costs are also an issue. Molecular markers are a convenient tool for rapid genetic analyses, allowing researchers to distinguish HMW-GS alleles from LMW-GS alleles. Many functional markers have been developed for glutenin loci, including a set of PCR markers, which was designed to distinguish allelic variations at the Glu-A3 locus [12]. Zhao et al. developed several functional markers to discriminate certain Glu-D3 and Glu-B3 haplotypes [13, 14]. Zhang et al. developed a new molecular marker system for identifying LMW-GS gene family members [8]. 

SDS sedimentation volume has long been used to characterize wheat flours and meals with the aim of predicting processing and end-product qualities [15–19]. Core collections of wheat germplasms are the minimum number of germplasm resources that can represent the maximum diversity of genetic resources within a species [20]. In this paper, we used a set of STS markers specific to the Glu-B3 locus to screen the core collections of China wheat in order to determine the distribution of Glu-B3 alleles of low molecular weight glutenin in the wheat core collections and provide information for wheat quality breeding.

## 2. Materials and Methods

### 2.1. Plant Materials

A total of 283 varieties were obtained from China wheat core collections including 152 landraces, 106 improved cultivars, and 25 introduced cultivars from three major growing zones including the spring wheat zone, the winter wheat zone, and the spring-winter wheat zone in China. All varieties were kindly provided by Crop Genetic Resources and Improvement, Institute of Crop Science, CAAS, China. 

### 2.2. DNA Extraction and PCR Amplification

Genomic DNA was extracted from seeds using the CTAB procedure as reported by Gale [21]. PCR was performed in a 10 *μ*L volume containing 20 ng of genomic DNA, 100 *μ*M of each dNTPs, 0.3 *μ*M of each primer (*glu-B3a*, *glu-B3b*, *glu-B3c*, *glu-B3d*, *glu-B3e*, *glu-B3fg*, *glu-B3g*, *glu-B3h*, *glu-B3i*, and *glu-B3bef*) [22], 0.1 U of Taq DNA polymerase (Trans), and 1 × PCR buffer (containing 2.5 mM MgCl_2_). PCR cycling conditions for gene-specific primers [22] were 5 min at 94°C followed by 38 cycles of 45 s at 94°C, 45 s at 56–61°C, 90 s at 72°C, and a final extension step of 8 min at 72°C. Amplified PCR products were separated on a 1.2% agarose gel.

### 2.3. SDS-Sedimentation Test

The SDS-sedimentation test was modified based on the method described before [19]. Here, we used 3.0000 g ground whole meal and 2% SDS-lactic acid liquid agent, the shaking time was 5 min, and the sedimentation was read after being settled for 5 min. 

### 2.4. Statistical Analysis

The analysis of variance (ANOVA) was performed for the 283 wheat germplasms to investigate the association between SDS-sedimentation volume and Glu-B3 alleles. The *R* ^2^ values obtained from ANOVA were used to represent the genetic effects of the Glu-B3 alleles on the SDS-sedimentation volume of wheat cultivars. ANOVA and *t*-test were performed using the SAS System (SAS Institute Inc., Cary, NC, USA).

## 3. Results and Discussion

### 3.1. Detection of Glu-B3 Alleles Using Specific PCR Primers

**Figure 1:**
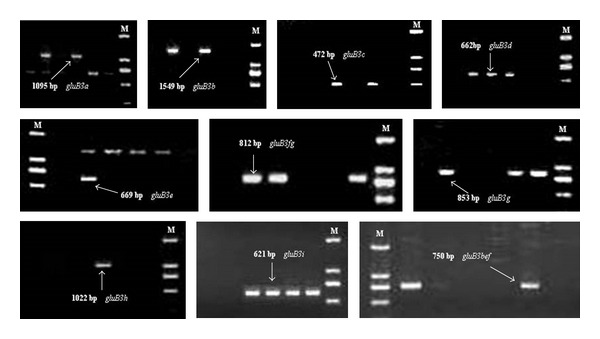
PCR products amplified from some varieties using 10 Glu-B3 allele-specific markers.

A total of 283 wheat varieties were screened using the 10 pairs of primers [22]. The Glu-B3a, Glu-B3b, Glu-B3d, Glu-B3e, Glu-B3 g, Glu-B3 h, and Glu-B3i alleles were successfully amplified in these cultivars. The expected target sizes were obtained, and the examples are shown in Figure 1. PCR had been proved to be the simplest, most accurate, lowest-cost method for identification of *Glu-A3* and* Glu-B3 *[7, 22] alleles in breeding programs. In the present study, the target bands were clear, and the results also indicated that this technique is effective. 

### 3.2. Influence of Glu-B3 Alleles on the SDS-Sedimentation Volume

The frequencies of the Glu-B3 alleles in the 283 cultivars and the SDS sedimentation volume are given in Table 1. The highest frequency was found for the Glu-B3i allele with 21.9%, followed by the Glu-B3a (13.8%) and Glu-B3g (13.8%) alleles. The lowest allele frequency was found for Glu-B3h (2.1%). The frequencies of alleles Glu-B3d, Glu-B3f, Glu-B3b, Glu-B3c, Glu-B3e, and Glu-B3j were 11.3%, 10.2%, 9.2%, 6.7%, 6.4%, and 4.6%, respectively. 

 The ANOVA analysis showed that Glu-B3 locus could predict 8.6% of the variance in wheat SDS sedimentation volume. Association analysis found that Glu-B3h, Glu-B3g, and Glu-B3b significantly heightened the SDS sedimentation volume, but Glu-B3c, Glu-B3j, and Glu-B3a significantly lowered the SDS sedimentation volume. The influence of other alleles on SDS sedimentation volume was not significant (Table 1) based on the total average SDS-sedimentation volume of the 283 cultivars.

Among landraces, Glu-B3b was found to significantly heighten the SDS sedimentation volume, but Glu-B3c, Glu-B3a, and Glu-B3j significantly lowered the SDS sedimentation volume. The influence of other alleles on SDS sedimentation volume was not significant (Table 2); however, for improved cultivars, not only Glu-B3b but also Glu-B3g and Glu-B3h significantly heightened the SDS sedimentation volume. Glu-B3i and Glu-B3j significantly lowered the SDS sedimentation volume. The influence of other alleles in improved cultivars on SDS sedimentation volume was not significant. Among the introduced cultivars, Glu-B3b, Glu-B3f, and Glu-B3g had significantly larger SDS sedimentation volume. 

Since the Glu-B3b had a more pronounced effect on gluten strength and dough development time [23] and the Glu-B3g had a good contribution to bread-making quality [24], varieties with Glu-B3b and Glu-B3g can be considered suitable germplasm for breeding new, high-quality cultivars. After a long selection based on the wheat quality, the most desirable alleles Glu-B3b and Glu-B3g became more and more popular in Chinese wheat, as indicated by 7.9% Glu-B3b in landraces, 12.3% in improved cultivars, 4.0% in introduced cultivars, and 9.9%, 14.2%, and 36.0% Glu-B3g in those wheats, respectively. However, the least desirable alleles Glu-B3a and Glu-B3c got less and less as indicated by 21.1% and 6.6% in landraces and 6.6% and 5.7% in improved cultivars, respectively, suggesting that wheat grain quality in Chinese wheat has been significantly improved through breeding effort.

 The LMW-GS and HMW-GS proteins are important parts of the gluten complex of wheat. They are encoded by a highly variable gene family. Because of their importance in wheat flour quality and the difficulties in discriminating them using traditional SDS-PAGE techniques, it was convenient to identify different LMW-GS alleles using allele-specific markers. We have characterized the distribution of the Glu-A3 and Glu-B3 loci alleles in the mini core collections of Chinese wheat germplasms [25]. If Glu-D3 locus allele-specific markers can be developed, this information will promote comprehensive understanding of the distribution of LMW-GSs in Chinese wheat germplasms and provide important references for breeding high quality varieties.

## 4. Conclusion

The LMW-GS proteins at Glu-B3 locus could predict 8.6% of the variance in wheat SDS sedimentation volume, the subunits Glu-B3b, Glu-B3g, and Glu-B3h could significantly heighten the SDS sedimentation volume, and Glu-B3a, Glu-B3c, and Glu-B3j could significantly lower the SDS sedimentation volume.

## Figures and Tables

**Table 1:** The Glu-B3 alleles and SDS-sedimentation volume.

Alleles	No. of accession	Frequency (%)	Mean SDS sedimentation	Range
Glu-B3a	39	13.8	25.2*	8.5–39.0
Glu-B3b	26	9.2	31.5*	19.3–47.0
Glu-B3c	19	6.7	24.2**	13.5–38.8
Glu-B3d	32	11.3	28.1	10.3–47.0
Glu-B3e	18	6.4	27.6	13.5–46.0
Glu-B3f	29	10.2	29.6	13.5–55.5
Glu-B3g	39	13.8	32.2*	16.5–61.3
Glu-B3h	6	2.1	35.3**	26.5–44.5
Glu-B3i	62	21.9	26.9	8.0–50.0
Glu-B3j	13	4.6	25.1*	16.5–35.5

*Significant at 5% probability level; **Significant at 1% probability level.

**Table 2:** Comparison of SDS-sedimentation volumes within Glu-B3 alleles from different kinds of cultivars.

Allele	No. of cultivars with allele			Mean SDS sedimentation		
	Landrace	Improved	Introduced	Landrace	Improved	Introduced
Glu-B3a	32	7	/	24.3**	29.4	/
Glu-B3b	12	13	1	30.8**	31.1**	45.0
Glu-B3c	10	6	3	21.7**	27.3	26.6
Glu-B3d	13	19	/	26.9	29.0	/
Glu-B3e	18	/	/	27.6	/	/
Glu-B3f	15	11	3	27.7	28.7	42.2
Glu-B3g	15	15	9	27.9	31.6**	40.4
Glu-B3h	1	2	3	26.5	34.9**	38.6
Glu-B3i	32	27	3	27.2	25.4**	36.0
Glu-B3j	4	6	3	24.9**	22.3**	31.0

*Significant at 5% probability level; **Significant at 1% probability level.
